# “We Thought We Were Prepared, but We Were Not”: Experiences from the Management of the Psychosocial Support Response during the COVID-19 Pandemic in Sweden. A Mixed-Methods Study

**DOI:** 10.3390/ijerph18179079

**Published:** 2021-08-28

**Authors:** Karin Hugelius, Sara Johansson, Helena Sjölin

**Affiliations:** 1 Faculty of Medicine and Health, Örebro University, 702 81 Örebro, Sweden; helena.sjolin@oru.se; 2 Creative Mammals, 413 27 Gothenburg, Sweden; sara@creativemammals.se

**Keywords:** COVID-19, mental health, psychosocial support, psychosocial response, crisis management

## Abstract

This study aimed to describe experiences of managing mental health and psychosocial activities during the first six months of the COVID-19 pandemic in Sweden. A national survey was answered by a non-probability sample of 340 involved in the psychosocial response. The psychosocial response operations met several challenges, mainly related to the diverse actors involved, lack of competence, and lack of preparations. Less than 20% of the participants had received specific training in the provision of psychosocial support during major incidents. The interventions used varied, and no large-scale interventions were used. The psychosocial response organizations were overwhelmed by the needs of health care staff and failed to meet the needs of patients and family members. An efficient and durable psychosocial response in a long-term crisis requires to be structured, planned and well-integrated into the overall pandemic response. All personnel involved need adequate and specific competence in evidence-based individual and large-scale interventions to provide psychosocial support in significant incidents. By increasing general awareness of mental wellbeing and psychosocial support amongst health professionals and their first-line managers, a more resilient health care system, both in everyday life and during major incidents and disasters, could be facilitated.

## 1. Introduction

During the first half of 2020, the COVID-19 pandemic entailed a major adaption for the Swedish health care system, both organizationally and for individual health care professionals. Intensive care units and residential care facilities for elderly people were under severe pressure, visiting restrictions were implemented, several groups of students switched to distance learning and the public faced anxiety, uncertainty and social restrictions ordered by authorities to reduce the spread of the virus [1]. The pandemic shared several characteristics common to other crises or disasters: a high degree of uncertainty, a dynamic and unpredictable course, and an imbalance between resources and needs [2,3]. However, a pandemic differs from other disasters by lasting longer and entailing risk for the individual health care professional regarding being infected or spreading the disease to a family member. Uncertainty has been described as the essence of stress [4]. The threat of a new, invisible virus can be harder to understand than other types of threats, increasing the level of uncertainty compared with other potentially traumatic events [5]. The mental health effects from the COVID-19 pandemic include pathologic conditions such as depression and anxiety [6,7] but also substantial negative mental health and wellbeing impacts such as loneliness [8], grief, and loss. Amongst health professionals, stress, worries, and emotional exhaustion have been reported [9], as well as mental health disorders such as post-traumatic stress disorder [10,11].

The pandemic caused a need for psychosocial response not only for primarily affected persons, infected patients, and their family members, but also for health care professionals [12,13]. In this study, psychosocial support was defined as practical, emotional, and social support provided by societal entities due to a serious incident [14]. The psychosocial response can contribute not only to restoring social and behavioral functioning, but also to identifying individuals in need of specific trauma interventions. Psychosocial responses to major crises or disasters should rely on evidence-based principles [5]. Reuniting separated family members with one another, facilitating contact and providing emotional support and practical help should be the utmost priority [15]. Due to the characteristics of the pandemic, innovative, or adjusted means of providing evidence-based psychosocial support have been required [16,17].

Providing this support, according to Swedish laws and regulations, is part of regional health care responsibilities [18], and organization and management differ from region to region. Scientific knowledge of how to organize, manage and (in practice) provide evidence based psychosocial support in long-lasting crises such as pandemics is limited [5]. Such knowledge is essential to facilitate a resilient service that is able to improve mental wellbeing amongst a significant number of affected people, prioritize amongst vulnerable groups, and adequately prepare individuals and organizations. Therefore, this study aimed to describe experiences of managing psychosocial support during the first six months of the COVID-19 pandemic in Sweden.

## 2. Materials and Methods

A mixed-methods study with a convergent parallel design [19] was conducted, analyzing quantitative and qualitative data gained from a web-based survey.

### 2.1. Study Setting

The COVID-19 pandemic reached Sweden at the beginning of March 2020. During the spring, the pandemic challenged the health care system, and most regional health care services were on high alert (level two out of three within the disaster contingency system), managing the situation in a crisis mode organizational structure. During the summer, the number of infected people decreased, but in late October, the curve rose again (second wave) and stayed at a high level. The data for this study was collected during the period of 10 December 2020 and 15 February 2021, between the second and third waves of the COVID-19 pandemic, covering the participants’ experiences from the first six months of the pandemic. This timing was chosen to catch the startup phase of the pandemic, but at the same time not interrupt or disturb ongoing operations.

### 2.2. Study Participants

A non-probability voluntary study sample was used. To be eligible, the participant had to have been involved in providing psychosocial support during the COVID-19 pandemic, either in a managerial position or as a psychosocial support provider. An anonymous web-based survey was distributed through the contingency management units in all 21 health care regions in Sweden to 20 county council administrations, the National Society for Hospital Chaplaincy and the Swedish Red Cross. Participation was voluntary and anonymous. Informed consent was obtained digitally within the survey following the display of the written study information.

### 2.3. Survey

The web-based survey (using a web application for protected research data) in Swedish was developed by two of the authors (KH and SJ). The study consisted of between 20 and 26 questions, depending on the answers given by the participants. Of these, four were open-ended questions regarding the experience relation between preparedness and authentic experience from major incidents or disasters, experienced challenges, and advantages of being involved in the response and recommendations for similar future situations. The survey was tested in a pilot test with five participants with experiences of psychosocial response in major incidents. A few questions were re-formulated after the pilot. Therefore, data from these five persons were not included in the study.

### 2.4. Analysis

The qualitative open-answer data were first analyzed, strongly inspired by inductive thematic analysis [20]. All answers from the open-ended questions were extracted and analyzed together, regardless of the actual survey question they referred to. First, all the data were read several times by all the authors. Since the statements and answers were quite short, the analysis continued by sorting the data into subthemes and themes (this was done by one of the authors, KH). Thereafter, informed by the findings in the qualitative data, the quantitative questions were analyzed with descriptive statistics (frequencies and percentages) and Chi-square analysis, using the SPSS statistical program (IBM SPSS Statistics for Windows, Version 27.0., IBM Corp., Armonk, NY, USA). A *p*-value of ≤0.05 was considered significant. Finally, the results from both the quantitative and qualitative analyses were interpreted in relation to each other. This process involved all three authors.

### 2.5. Ethical Considerations

Permission to conduct the study was given by the Swedish Ethical Review Authority (dnr 2020-05796).

## 3. Results

### 3.1. Demographics

A total of 340 people participated in the study. Their demographic information and experiences are shown in Table 1. Half the participants had previous experience of being involved in psychosocial response services in major incidents, including bus accidents, major traffic accidents, fires and terrorist attacks, but no long-lasting situations. Most study participants had been deployed in a city, defined as a town with over 50,000 inhabitants, while 20% (*n* = 69) had been working in a metropolitan area (Stockholm, Gothenburg or Malmo), and another 20% (*n* = 68) had been deployed in a town or rural area. About 15% (*n* = 50) of all the participants had been involved in psychosocial response operations covering the whole country, such as the national general medical telephone services or a specific COVID-19 psychosocial support hotline. Most participants had been involved in the psychosocial response within the public health care services (*n* = 159, 47%), occupational health care services (*n* = 67, 20%) or social services (*n* = 66, 19%) (see Table 1).

Most participants had been involved in the psychosocial response as crisis support providers (*n* = 206, 61%), but participants who had been acting in a managerial position (39%, *n* = 134) were also represented (see Table 1). The majority had been involved in crisis support services as part of their ordinary duties (*n* = 193, 57%) (see Table 1).

Three themes emerged from the qualitative analysis: organizational issues, professional issues and contextual issues. Within these, the data were analyzed from the perspectives of positive experiences (facilitators) and negative experiences (barriers) for enabling an effective and resilient psychosocial response (main theme). Consequently, the subthemes (see Table 2) could include both facilitator findings and barriers. These and the quantitative findings that were analyzed in relation to the qualitative findings are presented and integrated under each theme. To ensure that no individual participant is identifiable, the quotations used in the results do not refer to a specific professional, gender or similar characteristic.

### 3.2. Organizational Issues

The many actors involved, including psychiatric care units, primary health care services, occupational health units, and hospital chaplaincy services, added challenges to the management and coordination of psychosocial support activities. This diversity caused a strong need for a clear organizational structure of the psychosocial response. Lack of coordination resulted in problems in analyzing needs, creating situational awareness and identifying gaps in psychosocial response services. Of the managers, 51% (*n* = 71) stated that psychosocial response services had been organized as an integrated part of the regional health care services’ crisis response organization. Many (45%, *n* = 63) were not sure, and a few (4%, *n* = 5) said they had not been integrated. Integration within the overall response facilitated the psychosocial response by adding structure and coordination. However, being part of a command-and-control structure required specific knowledge of the staff methodology and terminology used within the overall response. A lack of such competence limited the possibility of being integrated in the overall response. One barrier to enabling an effective response was the overall medical commanders’ lack of understanding of the importance of addressing mental health needs and using evidence-based principles for psychosocial support, which sometimes caused frustration amongst the managers for the psychosocial response operations.

Of all 340 participants, 21% (*n* = 72) reported that there had been an alignment and plan in place for the psychosocial response operations alongside the health care service while 29% (*n* = 98) said there had not been, and 48% (*n* = 164) were not sure. One informant said:

“There was a plan for how to create more beds in the hospital and how to send patients or health care professionals to other regions but no plan for how to provide psychosocial support, neither for family members nor for health professionals. Why didn’t we make a plan? I don’t know”.

In towns, rural areas or national districts, plans for the psychosocial support response were significantly more absent than in metropolitan areas or bigger cities (Chi-square test: plan available for metropolitan areas/cities, 33% vs. town/rural areas/national district, 11%, *p* = 0.001). One major challenge was having access to a sufficient number of competent crisis support personnel over time. Related to this, the diversity of formal training and methods used caused confusion about what kind of support was actually provided and how to distribute personnel to be as effective as possible.

### 3.3. Professional Issues

Few participants had received any kind of formal training on psychosocial support in major incidents. About half of the professionals had previous authentic experiences from psychosocial responses in major incidents (see Table 3), but most participants (*n* = 208, 61%) had no specific formal training on how to manage or provide psychosocial support in major incidents. Managers had received more training than providers (managers 52% (*n* = 70); providers, 30% (*n* = 139), *p* = ≤ 0.05). Participants deployed in a metropolitan area or city had more often received specific training than participants deployed in a town, rural area, or national district (Chi-square test: 39% in a metropolitan area or city vs. 27% in a town, rural area or national district, *p* = 0.001).

In particular, staff from occupational health care services had not been involved in either preparations or psychosocial response in major incidents and therefore lacked insight into both the organization of the overall response and the psychosocial response. Also, several managers expressed a general lack of knowledge of how the Swedish medical health care system responded to crises and the working methods used. This caused frustration and limited effective coordination of the response. One informant explained:

“The regional health care services’ internal organization was unclear to me. I would like to learn how to work in the crisis response organization. Some terms used were unknown to me. Although I know much about providing psychosocial support, a lot of other things were unclear”.

Some health care professionals, such as the nurses deployed in the national helpline for medical advice, felt unprepared and lacked basic knowledge on psychological trauma, crisis reactions, and psychosocial support principles. Also, formal structures, such as where to refer affected people in need of further assessment or individual support, were unclear and caused frustration amongst the nurses answering such phone calls.

Although many of the participants were professional psychologists, social workers, or behavioral scientists, many lacked the specific skills needed to provide psychosocial support in major incidents. This caused a lack of competent staff and reduced the possibility of offering evidence-based responses.

“Many of those who provided psychosocial support did not have specific competence in providing psychosocial support in such situations or in psychotraumatology, but more a general emotional supportive approach. Of course, we needed them as well, but we needed personnel with specific psychosocial support skills the most, not general emotional support”.

A lack of updated knowledge on evidence-based methods of providing psychosocial support was experienced, both amongst colleagues involved in the psychosocial response and among external personnel, such as within the overall response organization.

“Lots of people at higher levels, including both medical doctors and social workers, lacked insight into modern ways to provide psychosocial support. They were stuck in old methods, such as psychological debriefing. That is not good, and we need to abandon methods that are not evidence-based, and in addition take resources from modern interventions. But it was like fighting against Goliath; some persons just made decisions …”

About 37% (*n* = 127) of the participants reported that no specific concept or method had been used for providing psychosocial support. Amongst those who had used or relied on any specific concept, psychological first aid was the most frequent concept used (47%, *n* = 95), followed by national guidelines on psychosocial support in major incidents (38%, *n* = 76), TENTS guidelines (21%, *n* = 43), and trauma-informed care (9%, *n* = 19). Amongst those who reported that any kind of group sessions had been used, most reported that they had not used any specific methods or concept (*n* = 14), some (*n* = 9) had used ethical reflection, debriefing or a similar method (*n* = 9), after action review (*n* = 6) or professional guidance (*n* = 3).

“As a social worker in the intensive care unit, I’m used to facing despair. But not despair and visiting restrictions at the same time. The pandemic was beyond our fantasies and preparations”.

Many participants stated that they had felt personally prepared to respond to sudden-onset events, such as bus accidents or terrorist attacks, but not for a long-lasting, complex situation, such as a pandemic. A mismatch between preparations and expectations was expressed, even amongst experienced professionals used to meeting people in distress.

### 3.4. Contextual Issues

The receivers of psychosocial support were not the target groups expected and planned for. The major recipients of psychosocial support were health care professionals (*n* = 233), followed by the public or family members of persons infected with COVID-19 (*n* = 152), health care services first-line managers (*n* = 125) and persons infected with COVID-19 (*n* = 97). Fifteen participants (4%) had experiences of providing psychosocial support to children and youths under 18 years old. A failure to reach, prioritize, and adapt psychosocial support services to vulnerable groups, patients and their loved ones was described.

“We had expected more family members in need of psychosocial support to come to the hospital as usual, but they didn’t show up. We could not reach them, actually, due to the visiting restrictions. But they showed up later within psychiatric care. That was a failure for us”.

Visiting restrictions added an extra psychosocial burden on the family members of hospitalized patients and limited the possibilities to offer psychosocial support. For the health professionals, the absence of family members caused an increased workload, but also ethical and moral distress when the health professional felt guilt and that they were caring for a body, rather than a human being. The restrictions caused harm in several situations, with several participants mentioning that these restrictions should have been better adapted for the most vulnerable groups.

“Since loved ones were not allowed to be present, it became difficult for them to participate in the care. They had extensive needs for support, but also for medical information that I could not provide. At the same time, the absence of family members affected health care professionals to a large extent. It was more demanding to care for an unknown human compared to caring for a person with personal attributes, history and a social context. We need to rethink this in the future. The visiting restrictions caused so many worries and anxiety amongst the family members …”

The context of the pandemic caused a need to adjust the traditional forms for providing psychosocial support to the context, demanding new ways to work, such as remote support or digital alternatives. The most used way to provide psychosocial support was through individual telephone contact, followed by face-to-face group sessions and individual digital interventions (see Table 4).

The large needs for psychosocial support amongst health professionals surprised many responders. Psychosocial support providers sometimes felt that their services covered for a lack of leadership and collegial support, and that many of the referred problems and needs could have been met by a more conscious leadership amongst health professional first-line managers.

“We thought we were prepared, but we were not. Not on the massive need for psychosocial support amongst the health professionals. After many years of lacking leadership, we had to pay the price now”.

Furthermore, several participants stated that the psychosocial support organization was unprepared to meet the demands of a long-lasting crisis. Since the preparations had mainly been focused on sudden onset incidents and events, acting over a long period, where the acute-, middle-, and long-term phases of psychosocial needs were present at the same time. Many of the needs amongst the health care professionals were not strictly caused by the COVID-19 pandemic, but by other general work-related or private problems, rather than symptoms of being traumatized or affected by the pandemic itself. This added complexity to the planning for and reporting on psychosocial support. Some participants specifically mentioned such issues:

“We need to find ways to support first-line managers over a longer perspective. A resilient leadership and stable leaders are essential. At first, we met many personnel who worried about being infected, and later many personnel who felt offended by their employer. Now, during the latest months, there are more fatigue problems or private, personal problems. The needs vary over time, and we need to be more proactive in every phase”.

To meet the needs amongst health professionals, a more proactive strategy, promoting self-caring strategies, and training first-line managers in how to support their staff and identify those in need of professional support were mentioned as important to enhance resilience of the psychosocial response.

“There should have been some kind of screening filter to identify those who actually needed professional support. All those who were referred to us [occupational health care service] were not in need of professional support, but of good leadership and collegial social support. That would have solved many problems”.

It was emphasized that the promotion of mental wellbeing in major incidents relied on an everyday footing.

“I was surprised by the attitudes amongst the first-line managers and their lack of knowledge and understanding of their own importance in building resilient health care professionals. At the same time, they did not seek advice or guidance. I think that we need to work with preventive measures and train the first-line managers to better acknowledge and support their health professionals over time”.

To boost solid grounds for healthy workplaces and good leadership within health care services in general, and especially amongst first-line managers, was described as essential preparation for mental wellbeing in future major incidents.

## 4. Discussion

The psychosocial response during the first six months of the COVID-19 pandemic in Sweden had several challenges. These were mainly related to the diversity of actors involved, diverse competence, and lack of individual and organizational preparations, such as training, structures and plans. Few participants had received specific training on psychosocial support in major incidents. To promote an effective and resilient psychosocial response, a clear organizational structure, specific skills, adapted interventions and an increased awareness of the psychosocial needs and evidence-based psychosocial response in such situations were needed.

The multiplicity of actors and professions involved in the psychosocial response required a clear structure and a plan of action from both a short- and long-term perspective. However, many participants reported a lack of such structures. Collaboration and coordination across the borders of disciplines and professions has been mentioned as an essential part of managing post-disaster psychosocial support [21]. The experience of a lack of plans and structure can be caused by an actual absence of such structures or be a consequence of vague communication on these matters to the personnel involved. A clear organizational structure with adequate management and leadership has been found to facilitate crisis response in other medical major incidents [2] and could be seen as key to an effective and resilient psychosocial response. Also, since psychosocial response should ideally be integrated as part of the overall response in emergencies [22], the management of psychosocial response in long-lasting or major incidents requires specific knowledge and training [21]. Professionals involved in the provision of psychosocial support after major incidents or disasters should have the necessary skills and competence to act in such situations [21,23]. Very few of the participants in a managerial position in this study had gained specific training, and a lack of such competence, such as knowing the terminology and working procedures used, was expressed. Collaboration and management of crisis responses can be facilitated by interagency training and preparations [24]. In this study, only 22% of the managers and 6% of the psychosocial support providers had participated in such preparations. To facilitate an overall understanding of the general response amongst psychosocial responders as well as an increased awareness of psychosocial response amongst other responders, training together seems to be an important preparation that might facilitate future psychosocial response. In addition, the results suggest that bigger cities had more preparations in place, including plans and specific training, compared to towns, rural areas and national districts. To minimize such differences, uniform preparations are required, and national standards and operational procedures should be considered.

The most common receivers of psychosocial support in this study were frontline health care professionals. Since psychosocial support to health professionals was a large part of the overall psychosocial response and at the same time essential for providing medical care to patients in need of medical care, strategies to promote health and mental wellbeing within these groups are of great importance from a societal perspective. Since the level of evidence on interventions that are beneficial in promoting the resilience and mental health of frontline workers in pandemics is lacking [25], the value of the support provided in this study is hard to evaluate. However, it should be noted that most providers participating in this study reported the use of interventions with insufficient evidence, such as psychological debriefing sessions [26]. This result indicates a strong need to ensure that both providers and managers of psychosocial responses are updated on the interventions and strategies recommended. However, since the participants declared that not all interventions were repeated for acute or traumatic experiences, but long-lasting psychosocial problems, a mix of professional skills and interventions might be useful in a long-lasting situation such as a pandemic.

Being mentally prepared to be exposed to severe stress and having access to self-caring strategies and an organizational culture that supports each other are essential for frontline responders and should be implemented as part of their basic training [27]. Pre-incident psychological interventions, such as resilience training, could be beneficial for health professionals deployed in pandemics [28]. A worrying finding was that first-line managers within the health care sector lacked knowledge on acute crisis reactions and how to support their staff. The reported need to further strengthen the overall leadership and awareness of how to promote mental wellbeing, both as a first-line health professionals’ manager and as a health professional individual, is in line with previous suggestions [25]. Strategies to enhance such general awareness and promote general resilience within the health care system in general are needed and could be achieved by introducing basic knowledge on crises reactions, traumatic stress and evidence based psychosocial support in in basic training of future nurses, physicians, psychologists or social workers. Targeted training for first-line managers within the health care system is also essential. Such general approaches would all value the care of individuals affected by potential traumatic events in everyday life [7].

Pandemics and associated protective measures, such as social isolation, can have considerable psychosocial impacts on individuals and communities in both the short and long term [28]. Promoting closeness and contact with family members is the most essential part of crisis support in everyday emergencies, war, natural disasters, and pandemic outbreaks [5]. Failure to adapt visiting restrictions and reach out to vulnerable groups, such as the family members of COVID-19 patients, was prominent in the present study. It is possible that with more specific training in psychosocial support in disasters and major incidents, adapted and proactive psychosocial support interventions (e.g., using protective gear or distanced inventions) could have been used to a further extent.

Since the COVID-19 pandemic affected the whole society, it is somewhat surprising that very few community-based interventions were reported to have been used. For most people affected by a major incident, promoting a sense of security and providing information, psychoeducation and social support from their normal social network will be sufficient [29]. Community interventions, such as workplace programs, school programs and media messages, have been recommended to enable recovery and mental health resilience amongst the public in major incidents and disasters [30]. Positive mental health effects from combining the provision of relevant pandemic information with stress management strategies, such as coping strategies, have been reported [28]. In this study, such strategies were rarely reported to have been used, at least as part of the psychosocial response. Since Sweden has limited experiences of major crises or disasters during the last century, awareness and experience of using such large-scale interventions might be limited, and these would be advisable for inclusion in psychosocial response professionals’ preparations in the future.

This study has added perspectives to inform theory building within the landscape of crises management as well as practical perspective of providing psychosocial support in major incidents such as a pandemic. One of the core questions raised must be why evidence informed guidelines and recommendations on how to manage and provide psychosocial support in major incidents has not been implemented to a larger extant. Also, it has raised several questions that deserve further scientific attention. Studies on knowledge and attitudes towards psychosocial support amongst clinical health professionals, psychosocial responders, and disaster managers would be of interest to further understand the tensions and gaps indicated in this study. Comparisons between national guidelines, disaster preparations, and strategies to promote mental wellbeing could also be of interest. The results support the suggestion to study interventions to address large groups, including remote interventions such as online resources [28]. Traditional crisis management theories, including interagency and interprofessional collaboration, leadership and models for organizational structures, need to be further explored from a psychosocial and mental health response perspective.

### Limitations

Since there were no official registers of professionals involved in the psychosocial response, either at a national or a regional level, one obvious limitation of this study is the use of non-probability study sampling. To ensure the study participants’ anonymity, no questions on the exact organization or region the participants represented were asked. Therefore, it is not known whether the results are representative of the whole country or of the entire psychosocial response. However, the 340 participants had a broad representation amongst different geographical areas, professions, and type of organizations.

Another limitation is the lack of conformity regarding how to define and describe, for example, psychosocial support response and key terms used in disaster response. This is a commonly mentioned limitation within disaster health research [31]. Both qualitative and quantitative data were used to capture experiences from a large sample size and at the same time personal experiences and reflections. The use of a mixed-methods approach has both advantages and limitations. In this study, the quantitative data provided an overview of psychosocial responses from both a management and provision perspective, while the qualitative data added depth and enabled further exploration of the topic. Although the qualitative data comprised short written statements, it added value to the study and gathered input from more participants than individual interviews would have made possible. Merged, both qualitative and quantitative data gave a concurrent interpretation of the results.

## 5. Conclusions

An effective, efficient and, resilient psychosocial response in a long-term crisis such as a pandemic requires that operations be formally structured, planned and integrated into the overall response. Psychosocial support should also rely on updated evidence, interventions and approaches adapted to large-scale situations. One cornerstone to achieving this is adequate and specific competence in how to both manage and provide psychosocial support in major incidents. Increasing the general awareness of psychosocial needs and psychosocial support amongst health professionals and their first-line managers is suggested to facilitate a more resilient health care system, both in everyday life and in major incidents or disasters.

## Figures and Tables

**Table 1:** Overview of demographics, type of deployment and previous experiences.

		Priest or Deacon *n* = 72	Nurse *n* = 61	Psychologist *n* = 46	Social Worker *n* = 41	Psychotherapist *n* = 37	Medical Doctor *n* = 24	Behavioral Scientist *n* = 22	Official Services Officer *n* = 11	HR Officer *n* = 6	Other * n = 20	Total N = 340
Gender n (%)	Female	43 (60)	58 (95)	34 (73)	35 (85)	17 (46)	5 (79)	16 (73)	0	6 (100)	14 (70)	228 (67)
	Male	29 (40)	3 (5)	10 (22)	6 (15)	20 (54)	19 (21)	6 (27)	11 (100)	0	6 (30)	110 (32)
	Missing data			2 (4)								
Age n (%)	18–65 years	58 (81)	58 (95)	44 (96)	39 (95)	37 (100)	19 (79)	17 (77)	8 (73)	3 (50)	14 (70)	297 (87)
	≥66 years	14 (19)	3 (5)	0	2 (5)	0	5 (21)	5 (23)	3 (27)	3 (50)	6 (30)	38 (11)
	Missing data			2 (4)								
Type of area n (%)	Metropolitan area	9 (13)	14 (23)	20 (44)	12 (29)	3 (8)	5 (21)	3 (13)	0	0	3 (15)	69 (20)
	City	20 (28)	18 (30)	23 (50)	19 (46)	29 (78)	14 (58)	9 (41)	3 (27)	0	6 (30)	141 (42)
	Town or rural area	20 (30)	11 (18)	3 (6)	7 (17)	5 (13)	5 (21)	323 (32)	8 (73)	6 (100)	2 (10)	68 (20)
	National	23 (32)	0	0	3 (7)	0	0	3 (14)	0	0	9 (45)	50 (15)
Role within response n (%)	Manager	2 (3)	27 (44)	29 (63)	16 (38)	124 (64)	19 (79)	5 (23)	6 (55)	4 (67)	3 (15)	134 (39)
	Provider	70 (97)	34 (56)	17 (37)	25 (61)	13 (35)	5 (21)	17 (77)	3 (27)	2 (3)	17 (85)	206 (61)
Type of deployment n (%)	Ordinary duties	43 (60)	26 (43)	36 (78)	33 (81)	26 (70)	12 (50)	9 (41)	6 (54)	0	2 (19)	193 (57)
	Temporary duties	9 (13)	32 (53)	10 (22)	5 (12)	11 (30)	12 (50)	5 (23)	2 (18)	6 (100)	5 (25)	97(29)
	Voluntary basis	20 (28)	3 (5)	0	3 (7)	0	0	8 (36)	3 (27)	0	12 (60)	49 (14)
	Missing data										1 (0)	

* e.g., teacher, police officer or retired military major.

**Table 2:** Overview of findings.

Main Theme	Facilitators and Barriers for Enabling an Effective and Resilient Response		
Themes	Organizational Issues	Professional Issues	Contextual Issues
Subthemes	Organizational structures and coordination	Competence in psychosocial support in major incidents	Long-lasting event
	Integration within the overall response	General knowledge of crisis management	Adaption of methods used
	Planning and endurance	Personal preparedness	Unexpected and multifaceted needs

**Table 3:** Overview of preparations.

Preparations	All Participants n (%) N = 340	Psychosocial Support Managers n (%) *n* = 134	Psychosocial Support Providers n (%) *n* = 206	*p*-Value Managers vs. Providers
Previous authentic experience of psychosocial responses in major incidents	170 (50)	77 (57)	93 (45)	0.059 *
Any formal training received	132 (39)	70 (52)	62 (30)	≤0.005
Training in management of psychosocial responses	49 (14)	40 (30)	9 (4)	≤0.005
Training in methods for providing psychosocial support	71 (21)	35 (26)	36 (17)	≤0.005
Training in staff methodology	38 (11)	35 (26)	3 (1)	≤0.005
Tabletop/seminar exercise	31 (9)	22 (16)	9 (4)	≤0.005
Simulation exercise together with other stakeholders such as the rescue services	61 (18)	49 (37)	12 (6)	≤0.005

*p*-value was calculated with the Chi-square test or if marked with * with Fisher’s exact test.

**Table 4:** Means of providing psychosocial support.

Means	*n* * (N = 224)
Individual telephone sessions	198
Face-to-face group sessions	102
Individual digital sessions	91
Physical presence (unappointed) of a psychosocial provider at workplaces	72
Coaching of first-line managers	64
Written communication on web pages	63
Individual digital chat sessions	34
Written information (other than on web pages)	31
Digital applications (apps)	12
Participation in media	12
Indirect provision of psychosocial support through training of other health professionals	11
Digital group sessions	8

* One participant could report one or more means used.

## Data Availability

The data that support the findings of this study are available from the corresponding author upon reasonable request.
